# Identification of Metabolomic Biomarkers for Endometrial Cancer and Its Recurrence after Surgery in Postmenopausal Women

**DOI:** 10.3389/fendo.2018.00087

**Published:** 2018-03-12

**Authors:** Yannick Audet-Delage, Lyne Villeneuve, Jean Grégoire, Marie Plante, Chantal Guillemette

**Affiliations:** ^1^Centre Hospitalier Universitaire (CHU) de Québec Research Center, Faculty of Pharmacy, Laval University, Québec, QC, Canada; ^2^Gynecologic Oncology Service, CHU de Québec, Department of Obstetrics, Gynecology, and Reproduction, Faculty of Medicine, Laval University, Québec, QC, Canada; ^3^Canada Research Chair in Pharmacogenomi, Laval University, Québec, QC, Canada

**Keywords:** metabolomics, endometrial cancer, blood-based biomarkers, recurrence, mass spectrometry

## Abstract

Endometrial cancer (EC) is the most frequent gynecological cancer in developed countries. Most EC occurs after menopause and is diagnosed as endometrioid (type I) carcinomas, which exhibit a favorable prognosis. In contrast, non-endometrioid (type II) carcinomas such as serous tumors have a poor prognosis. Our goal was to identify novel blood-based markers associated with EC subtypes and recurrence after surgery in postmenopausal women. Using mass spectrometry-based untargeted metabolomics, we examined preoperative serum metabolites among control women (*n* = 18) and those with non-recurrent (NR) and recurrent (R) cases of type I endometrioid (*n* = 24) and type II serous (*n* = 12) carcinomas. R and NR cases were similar with respect to pathological characteristics, body mass index, and age. A total of 1,592 compounds were analyzed including 14 different lipid classes. When we compared EC cases with controls, 137 metabolites were significantly different. A combination of spermine and isovalerate resulted in an age-adjusted area under the receiver-operating characteristic curve (AUC_adj_) of 0.914 (*P* < 0.001) for EC detection. The combination of 2-oleoylglycerol and TAG42:2-FA12:0 allowed the distinction of R cases from NR cases with an AUC_adj_ of 0.901 (*P* < 0.001). Type I R cases were also characterized by much lower levels of bile acids and elevated concentrations of phosphorylated fibrinogen cleavage peptide, whereas type II R cases displayed higher levels of ceramides. The findings from our pilot study provide a detailed metabolomics study of EC and identify putative serum biomarkers for defining clinically relevant risk groups.

## Introduction

Endometrial cancer (EC) is the sole gynecological neoplasm with a rising incidence and mortality and is currently the most common gynecological cancer in the United States, Canada, and other developed countries (1). Occurring predominantly in postmenopausal women, EC is initially treated by surgery including total hysterectomy, bilateral salpingo-oophorectomy, and lymph node evaluation. Despite successful surgery, 10–15% of tumors recur within 5 years with poor treatment outcomes and low survival rates (2).

Predictive and prognostic factors of EC include histological subtype with high-risk features such as high-tumor grade, stage, and deep myometrial invasion (3). Endometrioid type I carcinomas and especially those of low grade exhibit a favorable prognosis and may be cured by primary surgery (4). Type II carcinomas include non-endometrioid histologies better represented by the prototypical serous carcinoma and account for ~10% of EC. Type II neoplasms represent higher-grade tumors with a more aggressive clinical course, for which recurrence is more frequent and treatment remains a challenge (5). Candidate diagnostic biomarkers such as CA125 and HE4 have been identified; however, their low sensitivity and/or specificity limit their use in the clinic (6, 7). ECs are strongly associated with cumulative estrogen exposure, obesity, and other characteristics of metabolic syndrome (8–12). This does not apply only to type I carcinomas, as type II tumors can also be associated with hormonal, reproductive, and metabolic factors (8). Based on the recognition of the biological and prognostic differences between pathogenetic types of EC and given the poor prognosis for recurrent disease, it is critical to develop novel biomarkers.

Metabolomics is defined as the comprehensive analysis of metabolites in a biological specimen and includes a more focused form of metabolomics that surveys lipids (referred to as lipidomics). This approach is becoming a very powerful tool for biomarker discovery and has proven itself useful in the study of many metabolic diseases including cancer. For instance, discoveries related to oncometabolites have highlighted the possibility of unsuspected cellular pathways whose components could serve as diagnostic or prognostic biomarkers or may be therapeutically targeted for disease treatment (13). Still, very few studies have used metabolomics in the context of EC (Table 1). In contrast, more than 30 global and targeted mass spectrometry (MS)-based metabolomics studies have been conducted for ovarian cancer and have identified dysregulated metabolic pathways that underlie several histological types of carcinoma (14). These discoveries have led to the identification of potential new therapeutic targets and biomarkers that might improve diagnosis and prognostication (15–18).

**Table 1:** Summary of previous metabolomics studies of EC.

Reference	Specimens	Platform (nb of metabolites)^a^	Upregulated metabolites	Downregulated metabolites
Trousil et al. (19)	Tissue from *n* = 10 controls	H^1^ NMR (68)	Valine, leucine, alanine, proline, tyrosine, phosphatidylcholine	Glutathione, scyllo-inositol, myo-inositol, inosine/adenosine
	*n* = 8 EC cases			

Shao et al. (20)	Urine from *n* = 25 controls	UPLC-QToF^b^	*N*-acetylserine, urocanic acid, isobutyrylglycine	Porphobilinogen, acetylcysteine
	*n* = 25 EC cases			
	*n* = 10 EH cases			

Gaudet et al. (21)	Serum from *n* = 250 controls	GC-MS (43)	None	C5-acylcarnitines, octenoylcarnitine, decatrienoylcarnitine, linoleic acid, stearic acid
	*n* = 250 EC cases			

Bahado-Singh et al. (22)	Serum from *n* = 60 controls	LC-MS/MS (181)	2-hydroxybutyrate, 3-hydroxybutyric acid, acetone, C10, C14:1, C14:2, C16, C18:1, C18:2, C2, C5-DC (C6-OH), C6 (C4:1-DC), C7-DC, C8, glutamate, SM C18:0	Asparagine, C3, histidine, hydroxyproline, kynurenine, l-methionine, lysoPC a C17:0, lysoPC a C18:0, lysoPC a C18:1, lysoPC a C18:2, methionine, several PC aa and PC ae^d^
	*n* = 56 EC cases	H^1^ NMR (32)^c^		

Troisi et al. (23)	Serum from *n* = 130 controls	GC-MS (259)	Lactic acid, homocysteine, 3-hydroxybutyric acid	Progesterone, linoleic acid, stearic acid, myristic acid, threonine, valine
	*n* = 118 EC cases			
	*n* = 30 OCa cases			
	*n* = 10 BED cases			

*EC, endometrial cancer; EH, endometrial hyperplasia; BED, benign endometrial disease; OCa, ovarian cancer; GC–MS, gas chromatography–mass spectrometry; H^1^ NMR, proton nuclear magnetic resonance; UPLC-QToF, ultra-performance liquid chromatography–quadrupole time-of-flight mass spectrometry; LC-MS/MS, liquid chromatography–tandem MS; PC, phosphatidylcholines; PC aa, diacyl-phosphatidylcholines; PC ae, acyl–alkyl-phosphatidylcholines*.

*^a^The number of metabolites examined is shown in parentheses*.

*^b^The authors did not report the number of metabolites detected*.

*^c^Metabolites detected by H^1^ NMR were also detected by LC-MS/MS*.

*^d^PC aa and PC ae were PC aa C36:0, PC aa C36:1, PC aa C36:3, PC aa C36:5, PC aa C36:6, PC aa C38:0, PC aa C38:5, PC aa C40:2, PC aa C42:2, PC aa C42:6, PC ae C34:0, PC ae C34:2, PC ae C34:3, PC ae C36:1, PC ae C36:2, PC ae C36:3, PC ae C38:0, PC ae C38:1, PC ae C38:2, PC ae C38:5, PC ae C38:6, PC ae C40:1, PC ae C40:6, PC ae C42:1, PC ae C42:2, PC ae C42:3*.

In the present study, our goal was to identify non-invasive biomarkers of EC cancer and recurrence in postmenopausal women using global metabolomics and lipidomics profiling. We examined preoperative serum metabolites in control women as well as those from women with EC of both histological types; these individuals were from a prospective study of women who underwent hysterectomy and were recruited from a single center. We used validated metabolomics platforms capable of identifying and quantifying multiple biochemical species simultaneously across all major metabolite classes as well as complex lipids including phospholipid, sphingolipid, and neutral lipid classes. In the first series of analyses, we identified putative EC biomarkers by comparing EC cases to control women. Then, we compared type I and type II EC cases to find biomarkers associated with histological type. In the third set of analyses, we compared matched recurrent (R) and non-recurrent (NR) cases of both histological type I and type II to detect recurrence biomarkers of EC. Finally, to identify putative biomarkers that would be specific to either type I or type II carcinomas, we compared matched R and NR cases of individual histological type.

## Materials and Methods

### Study Population

All participants provided written informed consent for their participation in the study and for the use of their specimens. The current study was evaluated and approved by the local Ethical Research Committee of the Centre Hospitalier Universitaire (CHU) de Québec—Université Laval (2012-993) and was conducted in accordance with the Declaration of Helsinki. The recruitment and specimen collection have been described (8). Briefly, participants were all recruited at the Hôtel-Dieu de Québec Hospital (Québec City, QC, Canada), between 2002 and 2014. All women were of postmenopausal status and underwent surgery (hysterectomy and bilateral salpingo-oophorectomy), either for EC treatment or for non-malignant conditions (*n* = 9 for benign pelvic mass, *n* = 3 for prophylactic treatment, *n* = 3 for precancerous cervical lesion, *n* = 2 for fibroma, *n* = 1 for uterine prolapse). Fasting blood samples were collected on the morning of surgery and were rapidly processed and stored at −80°C until analysis. To be eligible, women must not have developed prior malignancies nor taken hormone replacement therapy (HRT) during the 3 weeks preceding specimen collection. EC recurrence was ascertained by computerized tomography scan and further confirmed by histopathology when required. Nurses collected information regarding demographic and anthropometric data through standardized questionnaires. A pathologist assessed the histopathological characteristics of the hysterectomy specimens for women with EC. Systematic compilation and review of medical records were performed by one of the treating gynecologic oncologists (Jean Grégoire), for both cases and controls.

Recurrent EC cases consisted of endometrioid (*n* = 12) or serous carcinoma (*n* = 6). To reduce potential confounding factors, NR EC cases were matched to R cases according to (i) histological type, (ii) grade, (iii) a body mass index (BMI) within an interval of 5 kg m^−2^, and (iv) age. In addition, (v) myometrial invasion was also considered for the matching of R and NR type I cases. We achieved a perfect match for the first two criteria for both histological types, as well as myometrial invasion for type I carcinomas. The median difference in BMI was 2.0 kg m^−2^. For two pairs of matched cases, the differences in BMI were of 7.2 and 11.2 kg m^−2^ (Table S1 in Supplementary Material). The median difference in age was 7 years. Control women were not matched to EC cases.

### Metabolomics

Serum sample aliquots were analyzed for metabolites and lipids with the metabolomics platform at Metabolon Inc. (Durham, NC, USA). Global profiling was conducted as described (24). Briefly, samples were prepared using the automated MicroLab STAR system (Hamilton Company, Reno, NV, USA). A recovery standard was added prior to the first step in the extraction process for quality control purposes. Metabolites were extracted by vigorous agitation after precipitation of proteins with methanol. Samples were then split to enable analysis by different methods, utilizing a Waters ACQUITY ultra-performance liquid chromatography (UPLC) system coupled to a Thermo Scientific Q-Exactive high-resolution accurate-mass spectrometer equipped with a heated electrospray ionization source and an Orbitrap mass analyzer. Raw data extraction, peak identification, and quality control processing were carried out using the Metabolon proprietary hardware and software. Compound identification was done through comparison with a library of chromatographic and MS data from authenticated standards.

Complex lipid profiling was conducted according to a modified version of a previously described protocol (25). Briefly, lipids were extracted from serum samples by a heptane/ethyl acetate mixture after addition of a butanol/methanol solution. Phase separation was induced by addition of aqueous acetic acid and centrifugation. MS analysis was conducted on a Shimadzu LC with nano PEEK tubing coupled to a Sciex SelexIon-5500 QTRAP. The scan was performed in multiple reaction monitoring mode. Peaks were quantified using the area under the curve (AUC) method, and data were normalized for inter-day signal differences. The analytical variability was ≤10% for both global profiling and lipidomics.

### Statistical Analyses

The similarity between groups (EC cases vs. controls, R vs. NR cases) was assessed by Student’s two-sample *t*-test for continuous variables, with correction for variance unequality when required (Welch’s two-sample *t*-test). Chi-square tests were used for categorical data or Fisher’s exact test when appropriate. Metabolomics data were log-transformed prior to statistical comparisons, and fold changes (FCs) were calculated based on the geometric mean. The Welch’s two-sample *t*-test was conducted for all comparisons, as it offers a slightly better statistical precision than paired sample analysis, which could have been used for the comparison of R to NR cases (26). Pathway enrichment analyses were performed with Metabolon online tools and using their proprietary database. The enrichment score was calculated by dividing the ratio of statistically significant metabolites within a pathway by the overall proportion of statistically significant metabolites.

Predicted probabilities, calculated with logistic regression, were used to build univariate and multivariate receiver operating characteristic (ROC) curves for EC and recurrence detection. Univariate regression models were made for each of the top four altered metabolites, and multivariate models were adjusted for age. BMI did not significantly contribute to the models (*P* > 0.80). Multivariate models were then optimized to give the best AUC with a maximum of four metabolites. The size of our group allowed the detection (α = 0.05, 1 − β = 0.80) of an AUC >0.700 for EC detection, and an AUC >0.730 for recurrence (27). Finally, because of the exploratory nature of the study, as well as the number of metabolites tested (*n* = 1,592), statistical adjustment for multiple tests was not performed.

## Results

### Markers of EC - Comparison of all EC Cases Relative to Controls

We examined preoperative serum metabolites from control women (*n* = 18) and from women with NR (*n* = 18) or R (*n* = 18) cases of either type I (*n* = 12 R and *n* = 12 NR) or type II (*n* = 6 R and *n* = 6 NR) carcinomas. To reduce biases that may be caused by menstrual cycling, all of the women were postmenopausal, and none of them had used HRT in the 3 weeks preceding specimen collection. All individuals were selected from a larger cohort recruited at a single center (8), and pairing of NR cases with R cases was based on pathological (histological type, grade, and myometrial invasion) and clinical (BMI and age) characteristics (Table 2; Table S1 Supplementary Material). A total of 1,592 compounds of known identity across all major metabolite classes, and 14 different lipid classes were assessed by UPLC–tandem MS using global profiling and lipidomics.

**Table 2:** Demographics of control postmenopausal women and those who were newly diagnosed with endometrial cancer (EC).

			EC cases (*n* = 36)			
Characteristic	Controls (*n* = 18)		Non-recurrent (*n* = 18)		Recurrent (*n* = 18)	
**Continuous variable data**	**Mean ± SD**		**Mean ± SD**		**Mean ± SD**	
Age (years)	58.9 ± 10.4^a^		66.3 ± 8.3		67.5 ± 9.4	
Height (cm)	159.2 ± 5.3		157.9 ± 5.4		156.5 ± 6.7	
Weight (kg)	70.1 ± 20.1		70.7 ± 16.9		68.3 ± 14.1	
BMI	27.5 ± 7.2		28.4 ± 7.0		28.0 ± 6.4	
Mean follow-up (months)	NA		56.3 ± 26.5		65.4 ± 48.7	

**Categorical data**	***n***	**(%)**	***n***	**(%)**	***n***	**(%)**
Full-term pregnancy						
No	4	(22)	7	(39)	8	(44)
Yes	14	(78)	10	(56)	9	(50)
Missing	0	(0)	1	(6)	1	(6)
OC use						
No	8	(44)	10	(56)	12	(67)
Yes	10	(56)	7	(39)	5	(28)
Missing	0	(0)	1	(6)	1	(6)
Smoking						
Never	12	(67)	11	(61)	13	(72)
Current	4	(22)	4	(22)	3	(17)
Ex-smoker	2	(11)	3	(17)	2	(11)
HRT						
No	14	(78)	10	(56)	11	(61)
Yes	4	(22)	7	(39)	6	(33)
Missing	0	(0)	1	(6)	1	(6)

*^a^Control women were slightly younger than EC cases (*P* < 0.05). No other significant differences were noted between cases and controls. No statistical differences were noted between R and NR cases. Student’s *t*-test was used for continuous variable data, with adjustment for variance inequality when required (Welch’s two-sample *t*-test). Categorical data were assessed using the chi-square test (χ^2^) or Fisher’s exact test, when applicable*.

*HRT, hormone replacement therapy; OC, oral contraceptive; BMI, body mass index; NA, not applicable*.

**Figure 1:**
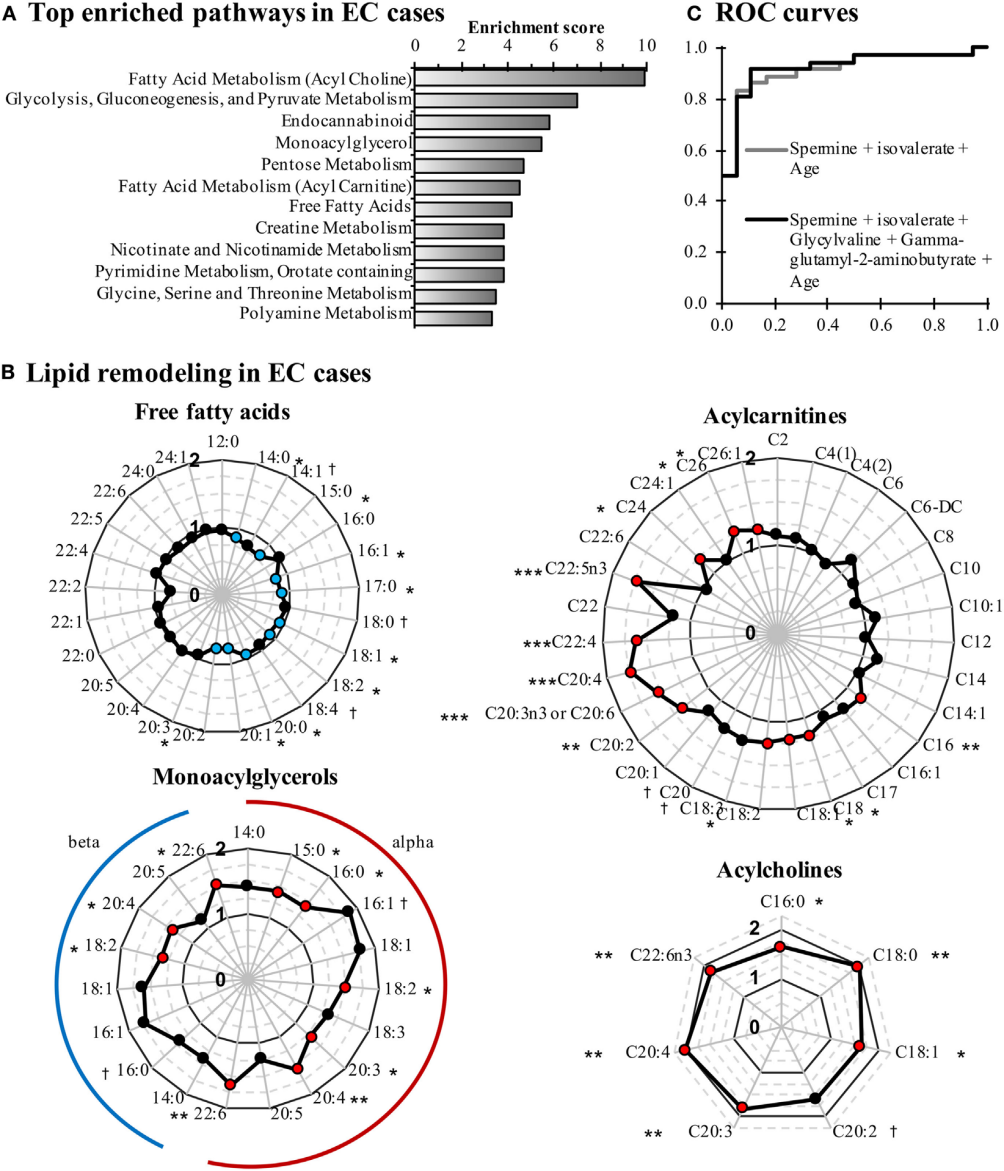
Comparison of endometrial cancer (EC) cases and controls reveals that lipid metabolism is perturbed in EC cases. **(A)** Pathway enrichment analysis is based on enrichment scores. Pathways containing at least three metabolites and having an enrichment score >3 are displayed. **(B)** Free fatty acid levels are lower in EC cases, whereas conjugated forms of fatty acids are elevated. Fold changes are displayed in radar graphs. Significantly enriched and depleted metabolites are marked in red and blue circles, respectively. **(C)** Receiver operating characteristic (ROC) curves of the most accurate regression models for detecting EC. ^†^*P* < 0.10, *P < 0.05, **P < 0.01.

When comparing EC cases to control women, 137 metabolites were significantly altered (115 up and 22 down, *P* < 0.05; Figure S1 in Supplementary Material). Pathway enrichment analysis identified lipid- and glycolysis-related pathways as the most affected in EC cases (Figure 1A). Conjugated forms of lipids, such as acylcholines, monoacylglycerols, and acylcarnitines, were generally higher in EC cases as compared with control women, whereas free fatty acids were detected at lower concentrations, supporting a remodeling of fatty acid metabolism in EC (Figure 1B). Of note, the C5 acylcarnitine 2-methylbutyrylcarnitine was also elevated in EC cases (FC = 1.27, *P* = 0.023). Five of the top 10 most altered features in EC cases were peptides and amino acids (Table 3), with spermine (FC = 7.66, *P* = 0.0004), and isovalerate (FC = –2.56, *P* = 0.015) as the most changed metabolites in cancer cases.

**Table 3:** Top 10 modulated preoperative serum metabolites in endometrial cancer (EC) cases.

Subpathway	Biochemical name	Fold change	*P*-value
**Biomarkers of EC**			

**EC cases (***n*** = 36) vs. control postmenopausal women (***n*** = 18)**			

Leucine, isoleucine, and valine metabolism	Isovalerate	−2.56	0.0154
Gamma-glutamyl amino acid	Gamma-glutamyl-2-aminobutyrate	−1.72	0.0170
Fatty acid, dicarboxylate	Adipate	−1.64	0.0456
Nicotinate and nicotinamide metabolism	1-methylnicotinamide	−1.47	0.0118
Histidine metabolism	Trans-urocanate	−1.45	0.0125
Methionine, cysteine, SAM, and taurine metabolism	Cystathionine	2.73	0.0011
Secondary bile acid metabolism	Isoursodeoxycholate	3.40	0.0146
Glycogen metabolism	Maltose	3.41	0.0005
Dipeptide	Glycylvaline	3.92	0.0075
Polyamine metabolism	Spermine	7.66	0.0004

**Biomarkers of EC histological types**			

**Type II EC cases (***n*** = 12) vs. type I EC cases (***n*** = 24)**			

Polypeptide	Bradykinin, des-arg(9)	−2.70	0.003
Androgenic steroids	Androsteroid monosulfate C19H28O6S	−2.33	0.030
Xanthine metabolism	1,3,7-trimethylurate	−2.33	0.047
Androgenic steroids	5-alpha-androstan-3beta, 17beta-diol disulfate	−2.17	0.025
Androgenic steroids	Androstenediol (3alpha, 17alpha) monosulfate	−2.08	0.017
TAG ester	TAG42:1-FA12:0	2.92	0.049
TAG ester	TAG46:3-FA18:3	3.05	0.030
TAG ester	TAG44:2-FA12:0	3.26	0.038
TAG ester	TAG44:2-FA18:2	3.38	0.041
Hemoglobin and porphyrin metabolism	heme	4.52	0.030

To further assess our ability to distinguish EC cases from controls based on these metabolites, we constructed ROC curves based on univariate and multivariate logistic regression models. The combination of spermine and isovalerate resulted in an area under the ROC curve (AUC) of 0.875 [95% confidence interval (CI) = 0.784–0.966], and an age-adjusted AUC (AUC_adj_) of 0.914 (95% CI = 0.833–0.994), very similar to a more complete model that included spermine, isovalerate, glycylvaline, and gamma-glutamyl-2-aminobutyrate and resulted in an AUC_adj_ of 0.921 (95% CI = 0.843–1.000) (Figure 1C). These results support the capacity of these metabolites to discriminate EC cases from controls.

### Markers Associated with Histological Types - Comparison of Type I and Type II EC Cases

A total of 98 metabolites significantly distinguished type I from type II ECs (*n* = 30 higher in type I, *n* = 68 lower in type I, *P* < 0.05). The most different metabolites between histotypes were bradykinin, with higher levels in type I (FC = 2.70, *P* = 0.003), and heme, which was 4.52-fold higher in type II ECs (*P* = 0.030) (Table 3). Levels of saturated long-chain acylcarnitines were higher in type II, with C20, C24, and C26 acylcarnitines displaying FCs of 1.32 (*P* = 0.021), 1.33 (*P* = 0.027), and 1.38 (*P* = 0.005), respectively.

Levels of choline were higher (FC = 1.27, *P* = 0.010) in type II ECs, along with sarcosine (FC = 1.42, *P* = 0.023), which are both metabolites of the tetrahydrofolate-serine/glycine pathway. Glycine levels tended to be elevated in type II ECs as well (FC = 1.23, *P* = 0.075). Levels of sulfated androgenic steroids differed significantly between the two histotypes, with type I EC having higher levels than type II for 10 out of the 18 androgenic compounds assessed by the method.

### Markers of EC Recurrence - Comparison of R and NR Cases of Both Histological Types

**Figure 2:**
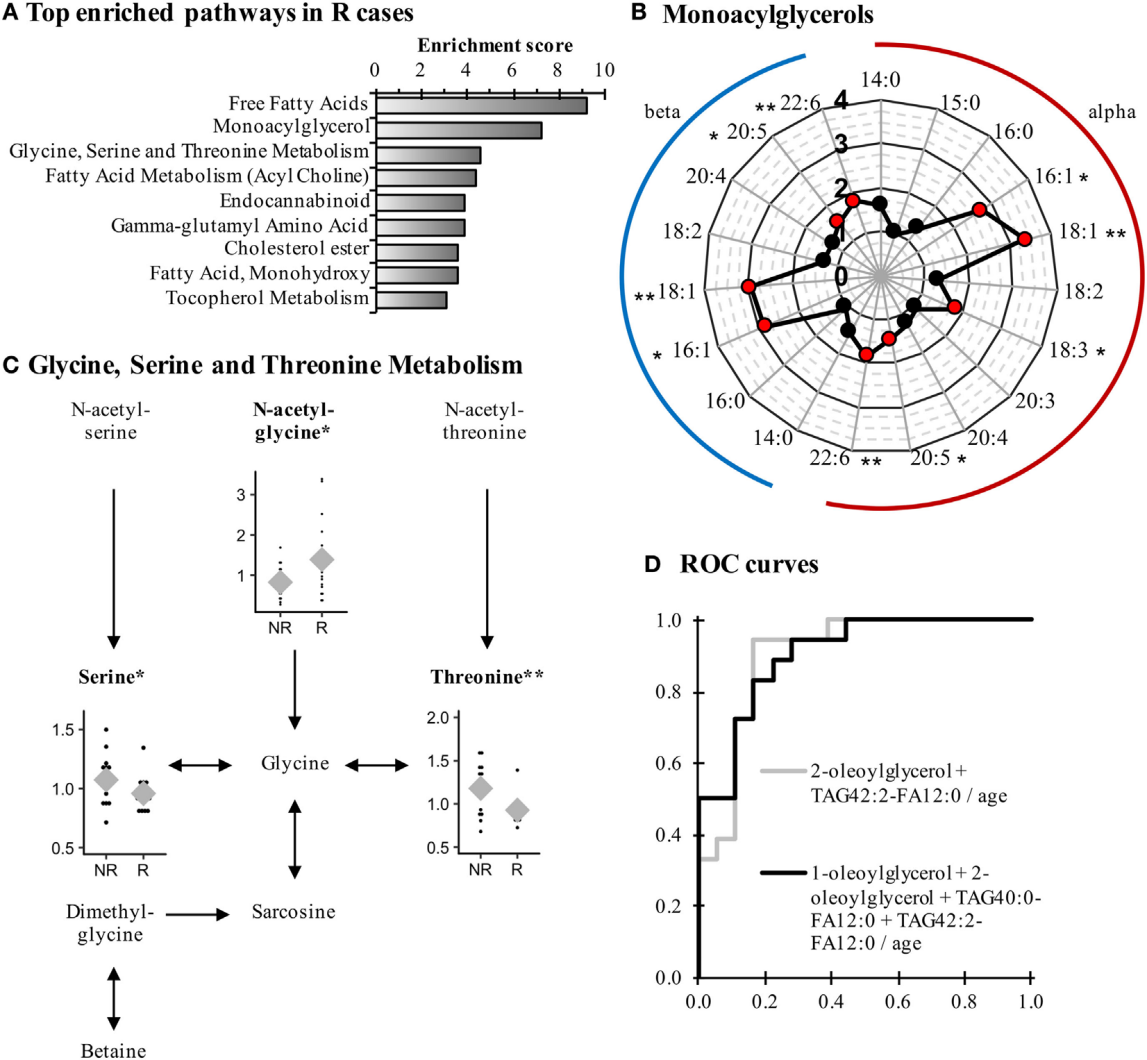
Monoacylglycerols and amino acids are remodeled in Recurrent (R) endometrial cancer cases when compared to Non-Recurrent (NR) cases. **(A)** Most-enriched pathways in R cases as compared with NR cases. Pathways containing at least three metabolites and having an enrichment score >3 are displayed. **(B)** Several species of monoacylglycerol are elevated in R cases. **(C)** The metabolism of glycine, serine, and threonine is perturbed in R cases in comparison to NR cases. Normalized levels of detected metabolites are displayed in dot plots, and means are represented by gray diamonds (♦). **(D)** Receiver-operating characteristics (ROC) curves of the most-accurate regression models to detect recurrence. *P < 0.05, **P < 0.01.

Recurrent cases were characterized by an altered lipid metabolism relative to NR cases. Among the 104 metabolites modulated, 80 represented lipid metabolism (68/75 up and 12/29 down; *P* < 0.05). Pathway enrichment analysis (Figure 2A) identified many classes of lipids affected in R cases, such as monoacylglycerols, for which 16:1, 18:1, 20:5, and 22:6 species of both alpha and beta isomers were significantly elevated (Figure 2B; Table 4).

**Table 4:** Top 10 modulated preoperative serum metabolites in recurrent endometrial cancer cases.

Subpathway	Biochemical name	Fold change	*P*-value
**Biomarkers of recurrence after initial surgery**			

**R cases (***n*** = 18) vs. NR cases (***n*** = 18) for type I and type II carcinomas**			

Ester	TAG40:0-FA12:0	−7.14	0.0427
Ester	TAG42:2-FA12:0	−5.00	0.0460
Primary bile acid metabolism	Chenodeoxycholate	−2.86	0.0263
Glycogen metabolism	Maltose	−2.78	0.0028
Secondary bile acid metabolism	Glycoursodeoxycholate	−2.00	0.0343
Fatty acid metabolism (acyl glycine)	Hexanoylglycine	2.04	0.0454
Monoacylglycerol	1-palmitoleoylglycerol (16:1)	2.73	0.0484
Monoacylglycerol	2-palmitoleoylglycerol (16:1)	2.86	0.0283
Monoacylglycerol	2-oleoylglycerol (18:1)	2.95	0.0076
Monoacylglycerol	1-oleoylglycerol (18:1)	3.37	0.0046

**Biomarkers of recurrence after initial surgery by histological type**			

**R cases (***n*** = 12) vs. NR cases (***n*** = 12) for type I endometrioid carcinomas**			

Secondary bile acid metabolism	Taurodeoxycholate	−7.14	0.0093
Secondary bile acid metabolism	Glycodeoxycholate	−4.55	0.0088
Primary bile acid metabolism	Taurocholate	−3.85	0.0383
Glycogen metabolism	Maltose	−3.45	0.0065
Primary bile acid metabolism	Glycocholate	−3.23	0.0263
Free fatty acids	FFA(22:5)	1.66	0.0006
Fibrinogen cleavage peptide	ADpSGEGDFXAEGGGVR	1.68	0.0135
Oxidative phosphorylation	Phosphate	1.76	0.0250
Ester	TAG58:10-FA20:5	1.88	0.0032
Monoacylglycerol	1-oleoylglycerol (18:1)	3.77	0.0450

**R cases (***n*** = 6) vs. NR cases (***n*** = 6) for type II serous carcinomas**			

Fatty acid metabolism (Acyl Carnitine)	3-hydroxybutyrylcarnitine	−2.17	0.0494
Pentose metabolism	Ribitol	−1.56	0.0192
Purine metabolism (Hypo)xanthine/inosine containing	Allantoin	−1.45	0.0481
Histidine metabolism	Histidine	−1.45	0.0028
Glutathione metabolism	2-aminobutyrate	−1.39	0.0132
Fatty acid metabolism (acyl choline)	docosahexaenoylcholine	2.18	0.0413
Monoacylglycerol	1-docosahexaenoylglycerol (22:6)	2.28	0.0036
Monoacylglycerol	1-oleoylglycerol (18:1)	2.29	0.0175
Phospholipid metabolism	Glycerophosphoinositol	2.38	0.0022
Monoacylglycerol	2-docosahexaenoylglycerol (22:6)	2.55	0.0067

In addition to modifications in lipid levels, other classes of compounds displayed significant alterations in R cases when compared with NR cases. For example, the pathway of glycine, serine, and threonine metabolism was affected, as both serine and threonine levels were lower in R cases, whereas the glycine precursor *N*-acetylglycine was elevated (Figure 2C). Even though higher levels of *N*-acetylglycine were observed, glycine was not affected, suggesting a rerouting of glycine metabolism intermediates in R cases.

Some metabolites had a similar association with recurrence in both type I and type II EC patients. This was the case of the monoacylglycerol 1-oleoylglycerol (18:1), the only metabolite observed among the top modulated metabolites for both histological types (Table 4), which displayed a FC of 3.77 (*P* = 0.045) and 2.29 (*P* = 0.018) for type I and type II, respectively. A similar observation was noted for other lipid metabolites, namely the acylcarnitine docosahexaenoyl carnitine (C22:6) (FC = 1.51 and 1.46 for type I and type II, respectively; *P* < 0.05) and the monohydroxylated fatty acids 2-hydroxypalmitate (2-OH-C16:0, FC = 1.47 and 1.57 for type I and type II; *P* < 0.05) and 2-hydroxystearate (2-OH-C18:0, FC = 1.30 and 1.56 for type I and type II; *P* < 0.05), suggesting a remodeling of lipid metabolism among R cases of both histological types when compared with NR cases. In addition, these four metabolites were not significantly different when comparing EC cases and control women (data not shown), suggesting their relevance as biomarkers of recurrence, independently of the histological type.

Receiver operating characteristic curves identified 2-oleoyl-glycerol and TAG42:2-FA12:0 as the most effective metabolites to discriminate R cases from NR cases, with an AUC = 0.877 (95% CI = 0.730–0.990) and an AUC_adj_ = 0.901 (95% CI = 0.796–1.000). These results are similar to the model including more metabolites, namely 1-oleoylglycerol, 2-oleoylglycerol, TAG40:0-FA12:0, and TAG42:2-FA12:0, which displayed an AUC_adj_ = 0.904 (95% CI = 0.807–1.000; Figure 2D), confirming the ability of these metabolites to predict recurrence.

### Markers Associated with Recurrence According to Histological Types - Comparison of R and NR Cases According to Histological Type

**Figure 3:**
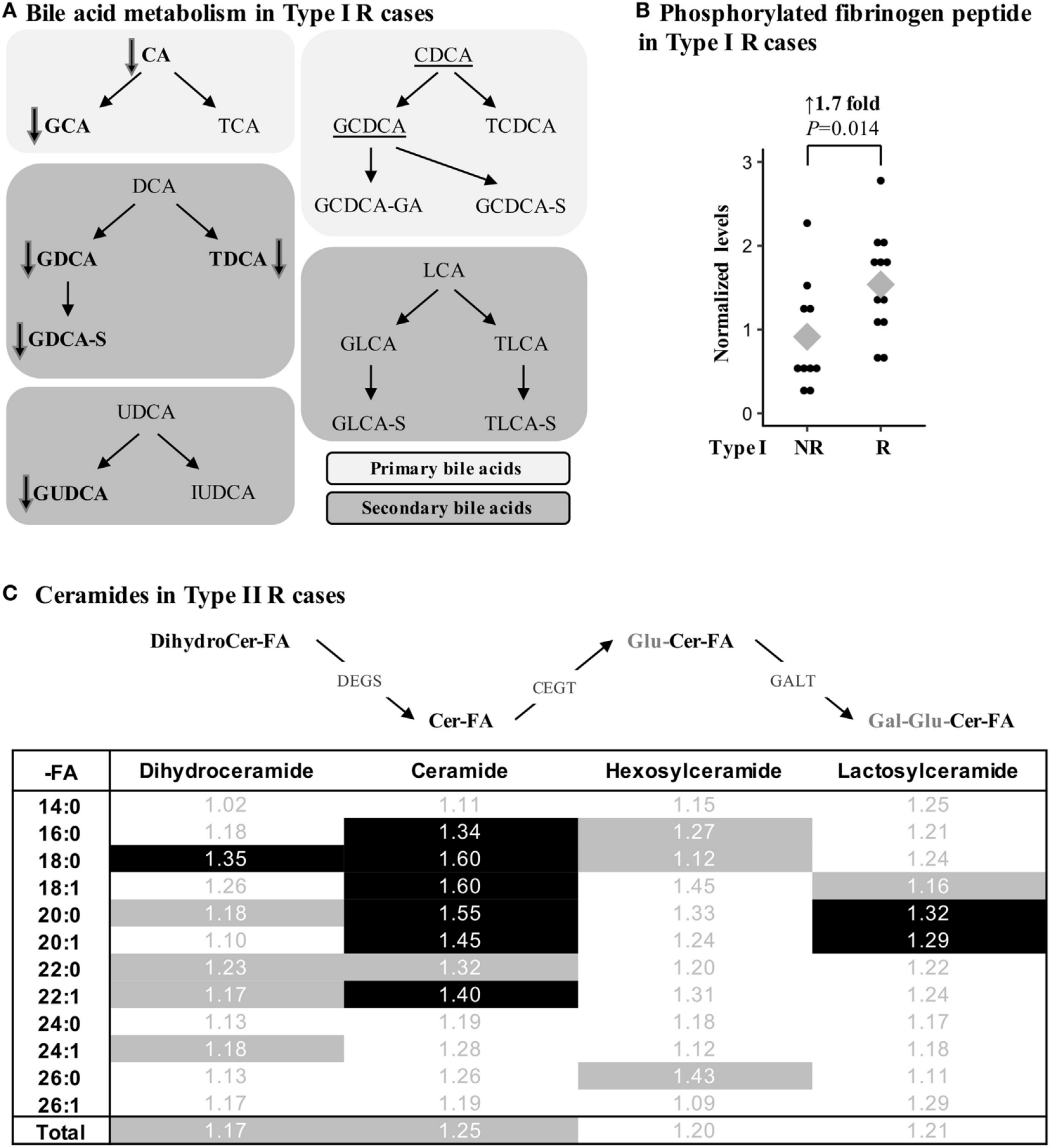
Metabolic alterations of Recurrent (R) endometrial cancer differ between the histological subtypes. **(A)** R cases of type I histology are associated with reduced bile acid levels. Metabolites in bold were significantly altered (*P* < 0.05), whereas a trend (*P* < 0.10) was detected for underscored metabolites. Bile acids can be conjugated with glucose (G-), taurine (T-), glucuronic acid (-GA), or sulfate (-S). CA, cholic acid; DCA, deoxycholic acid; UDCA, ursodeoxycholic acid; IUDCA, isoursodeoxycholic acid; CDCA, chenodeoxycholic acid; LCA, lithocholic acid. **(B)** Normalized levels of the phosphorylated fibrinogen cleavage peptide ADpSGEGDFXAEGGGVR were higher in type I R cases. **(C)** Ceramide levels were significantly altered in type II R cases. Fold change is shown, and significant metabolites (*P* < 0.05) are identified by a black background, whereas a trend (*P* < 0.10) in metabolite differences is shown by gray shading. Cer, ceramide; -FA, fatty acid group; DEGS, dihydroceramide desaturase; CEGT, ceramide glucosyltransferase; GALT, galactosyltransferase; Gal, galactose; Glu, glucose.

Several metabolites were specifically associated with recurrence in type I endometrioid or type II serous cases. For instance, modifications in bile acid metabolism were mainly observed in type I R cases, which had lower levels of primary and secondary bile acid metabolites such as taurodeoxycholate (FC = –7.14, *P* = 0.009), glycodeoxycholate (FC = –4.55, *P* = 0.009), and taurocholate (FC = –3.85, *P* = 0.038; Figure 3A; Table 4). Type I recurrent cases were also characterized by an enrichment in circulating levels of phosphorylated fibrinogen cleavage peptide ADpSGEGDFXAEGGGVR (FC = 1.68, *P* = 0.014; Figure 3B; Table 4); an association not found for type II R cases (*P* = 0.477).

In contrast, multiple classes of sphingolipids were significantly enriched in type II R cases, including ceramides, their precursors dihydroceramides, and their glycosylated derivatives lactosylceramides. Though the variations were modest with FCs between 1.29 and 1.60 for significant metabolites, numerous metabolites of this pathway were similarly altered, underscoring the potential significance of these variations (Figure 3C). None of the ceramides were significantly different in type I R cases, suggesting that alterations in these pathways could be specific to type II R cases.

## Discussion

In this study, we profiled 1,592 compounds in 54 postmenopausal women. To the best of our knowledge, this is the first study reporting metabolites associated with type I and type II EC carcinomas and their recurrence following initial surgical treatment. Our findings represent an important pilot study in the identification of putative serum biomarkers useful for detecting EC and predicting recurrence following initial surgery, to ultimately improve patient survival based on better stratification and informed treatment decisions.

We found that the levels of free fatty acids linoleic acid (C18:2) and myristic acid (C14:0) were lower in EC cases as compared with control women, consistent with previous reports comparing EC cases and controls (21, 23). Gaudet et al. (21) also observed modifications in intermediates from the branched chain amino acid pathway, such as isovalerylcarnitine/2-methylbutyrylcarnitine (undistinguishable by the MS method), which were also altered in our set of EC cases compared with controls. Other comparisons between the two studies could not be extended, as their panel of metabolites was targeted to 69 compounds. In our dataset, additional metabolites related to amino acids were affected in EC cases, such as polyamines, which are involved in cancer progression, including endocrine-related neoplasms like breast cancer (28). Accordingly, the most elevated metabolite between EC cases and controls was spermine, a biomarker of EC possibly originating from EC cells. This is conceivable as polyamine synthesis and degradation are actively regulated in the endometrium, notably during the menstrual cycle and pregnancy (29–31).

Lipids were also considerably affected in EC cases when compared with controls, with lower serum concentrations of free fatty acids but higher levels of conjugated fatty acids such as acylcholines, acylcarnitines, and monoacylglycerols. This is consistent with a report from Bahado-Singh et al. (22). They observed higher acylcholine levels in EC patients. However, very little is known about these lipids (no entries were found in either the Human Metabolome Database or Kyoto Encyclopedia of Genes and Genomes database; accessed on December 15, 2017), although some acylcholines, including palmitoylcholine, stearoylcholine, and oleoylcholine, enhance estradiol penetration through tissues (32). This could be important in the context of a hormone-sensitive cancer, potentially favoring the estrogenic activity of estradiol in the tumor. In addition, acylcarnitines have been linked to EC, as well as to breast cancer, where they are enriched in hypoxic tissues (21, 33). Acylcarnitines are synthesized by cells to fuel mitochondrial fatty acid oxidation. However, the mechanisms by which they are found in circulation remains unclear, although their levels should reflect cellular activity and concentration (34, 35). It is thus possible that circulating levels of acylcarnitines could reflect the hypoxic status of tumor cells.

We also observed an accumulation of monoacylglycerols to the detriment of free fatty acids. Monoacylglycerols are mainly derived from enzymatic hydrolysis of triacylglycerols and diacylglycerols and can be further metabolized to free fatty acids through the action of monoacylglycerol lipase (MAGL), an enzyme previously identified to be downregulated in EC (36). Accordingly, a lower MAGL activity could explain, at least in part, the observed accumulation of monoacylglycerols in sera of EC cases as compared with controls. Of note, the monoacylglycerol 1-oleoylglycerol (18:1) was strongly elevated in R cases of both tumor types and could represent a marker of EC recurrence. This is consistent with a role for MAGL in various aspects of tumorigenesis (37).

Modifications in lipid levels could also be related to bradykinin, a putative biomarker of type I EC that is known to activate phospholipase D in EC (38). As an inflammatory mediator, bradykinin triggers kinin-activated pathways. These have been associated with EC and breast cancer progression, supporting the role of bradykinin in tumors originating from steroid sensitive tissues (39, 40). Sulfated androgens were also higher in type I EC cases, consistent with the reported implication of sulfated steroids in this histotype (8, 41–44). Furthermore, our data identified heme as a putative biomarker of type II EC and highlighted modifications in pathways closely related to heme synthesis, namely the tetrahydrofolate-serine/glycine pathway (45). Heme consumption might participate in endometrial carcinogenesis, being associated with a moderate increase in EC risk (46). Targeting this pathway is currently being tested for the treatment of ovarian cancer and other solid tumors using a new drug, 4-(N-(S-penicillaminylacetyl)amino) phenylarsonous acid (PENAO), acting through the induction of heme degradation by heme oxygenase-1 (47, 48).

Putative biomarkers of EC recurrence were also identified. Compared with patients with non-recurrent type I carcinomas, those who experienced recurrence after surgery presented alterations in bile acid levels. Bile acids contribute to cholesterol homeostasis, a precursor of the steroids that drive the development and progression of this histological type of EC (10). Recently, we showed that higher levels of circulating steroids are linked to an increased risk of recurrence (43). Numerous enzymatic pathways are involved in the conversion of both bile acids and steroids, including reduction by aldo-keto reductases (49), conjugation by uridine diphospho-glucuronosyltransferases (50), sulfotransferases (51), and sulfatase (52). The reduced levels of bile acids may reflect an altered activity of some of these metabolic pathways in R type I cases, consistent with previous findings (8, 42). Bile acids might also act synergistically with steroids by stimulating EC cell growth, as they enhance myometrium sensitivity to hormones such as oxytocin (53). Finally, bile acids might initiate signaling events, as some of them display inflammatory functions (54, 55). In accordance with modifications in the inflammatory status of R type I cases, inflammatory response markers such as the phosphorylated cleavage peptide of fibrinogen were elevated in these patients. This is reinforced by studies that have linked the overexpression of procoagulants with gynecologic malignancies including EC, and further associated this overexpression with more aggressive tumor types (56–60). Although fibrinogen may confer a potential advantage to cancer cells in terms of aggressiveness and dissemination, the underlying mechanisms remain unclear.

For the type II ECs analyzed here, all of which were serous carcinomas, our observations revealed enhanced concentrations of numerous ceramides in preoperative sera of R cases compared with NR cases. Others have linked alterations of sphingolipids and ceramides in EC with the differentiation status of the tumors, but they did not include type II carcinomas (61–64). Tanaka et al. (65) showed that serous ovarian cancers exhibit elevated levels of glycosylated ceramides, consistent with high expression of galactosyltransferase in tumors. As ovarian serous cancers share similarities with type II serous EC, this raises the possibility that alterations in ceramide metabolism may be common to both tissues (66, 67). These bioactive lipids participate in tumor progression and the metastasis process (68) and, therefore, may represent promising biomarkers for non-invasive detection of recurrent type II EC. However, their metabolism in endometrial malignant tumors has been poorly characterized and our investigation is the first to present complex data on ceramide metabolism in the context of EC. Additional studies are thus warranted.

We identified putative cancer-specific and recurrence biomarkers using an unbiased metabolomics approach for type I and type II EC in postmenopausal women. Although exploratory, our study has several strengths including the analysis of postmenopausal cases and controls, as well as R and NR cases of two of the most common histological EC subtypes, in addition to the quantification of an extensive panel of metabolites through a validated metabolomics platform. This approach is powerful for screening a large and diverse set of metabolites but is limited in terms of absolute quantification. Additional limitations to our study include a restricted number of prospective EC cases, whereas the study design likely reduced variations through sample matching. Even though enrolled women must have not taken HRT during the 3 weeks prior to blood draw, it is not known if this period is sufficient to fully restore circulating metabolite levels potentially affected by HRT. Nonetheless, HRT use was similar between groups, which likely reduced the potential bias it might have introduced. The putative biomarkers identified in this pilot study will require validation in larger cohorts using quantitative methods. Their specificity to EC must also be confirmed, notably in comparison to other gynecological malignancies (ovarian cancer, mixed Müllerian cancer) and benign conditions (hyperplasia, endometriosis, etc.), which will facilitate their translation to the clinic. Finally, mechanistic studies are needed to help gain insights into the underlying biological processes driving the observed changes in metabolites in EC cases and those experiencing recurrence after surgery for curative intent.

## Ethics Statement

The study was evaluated and approved by local Ethical Research Committees of the Centre Hospitalier Universitaire (CHU) de Québec—Université Laval (2012-993). All subjects gave written informed consent in accordance with the Declaration of Helsinki.

## Author Contributions

CG designed and supervised the research. JG and MP were involved in patient recruitment. JG established the clinical database. LV prepared biospecimens. YA-D performed statistical analysis. CG and YA-D took part in the analysis and interpretation of the data and wrote the manuscript. All authors critically reviewed and approved the final version of the manuscript.

## Conflict of Interest Statement

The authors declare that there are no actual or potential conflicts of interest that could inappropriately influence, or be perceived to influence, this work.

## Abbreviations

EC, endometrial cancer; R, recurrent; NR, non-recurrent; MS, mass spectrometry; UPLC-MS/MS, ultra-performance liquid chromatography–tandem mass spectrometry; ROC, receiver operating characteristic; AUC, area under the curve; MAGL, monoacylglycerol lipase; PENAO, 4-(*N*-(S-penicillaminylacetyl)amino) phenylarsonous acid.
