# Supplementary material for: Identification of Metabolomic Biomarkers for Endometrial Cancer and Its Recurrence after Surgery in Postmenopausal Women
Source: Front Endocrinol (Lausanne). 2018 Mar 12;9:87. doi: 10.3389/fendo.2018.00087 (PMC5857535; doi:10.3389/fendo.2018.00087)
Supplement: Supplementary file 1 [file data_sheet_1.PDF]

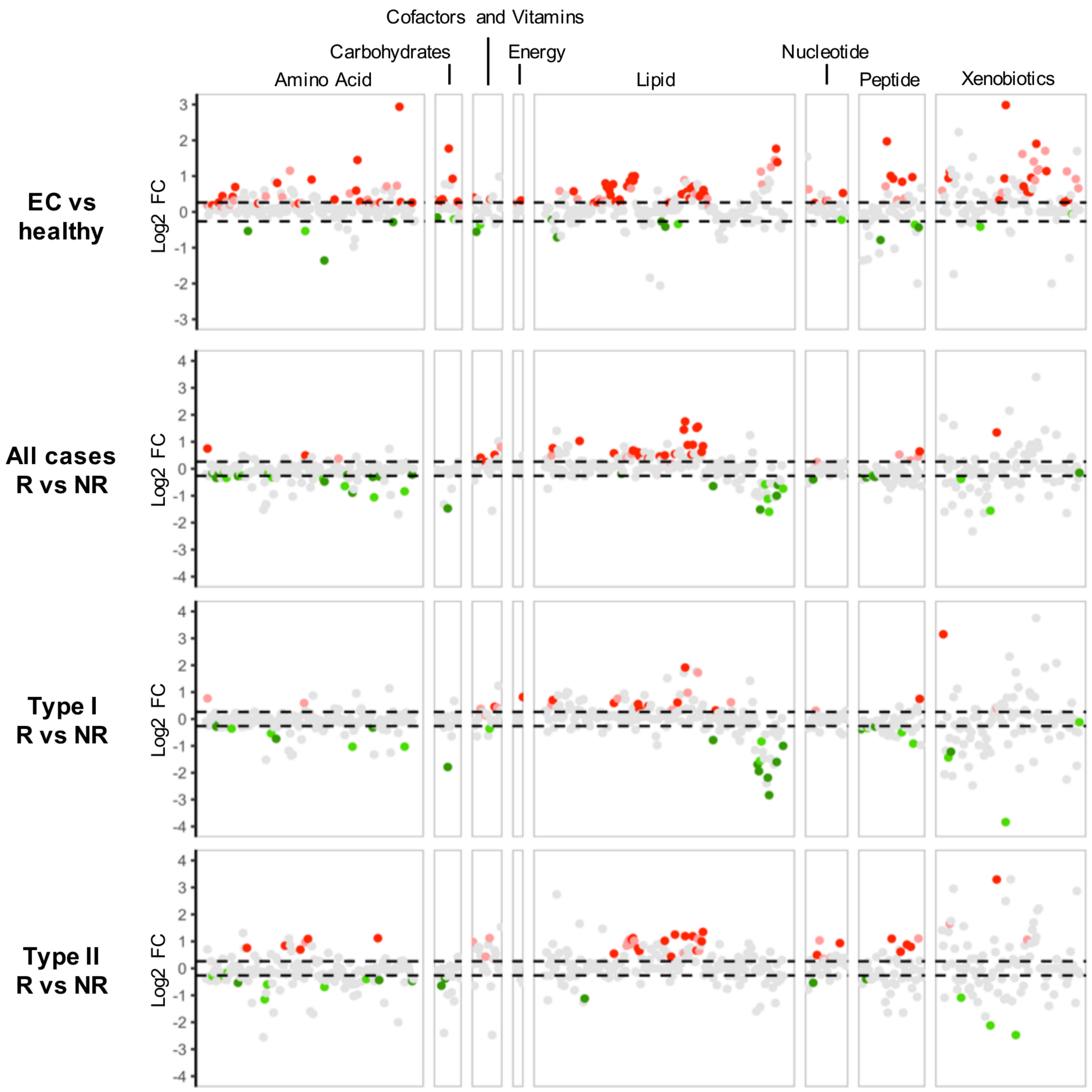

Supplemental Figure 1. Fold change (FC) of metabolite concentrations for endometrial cancer (EC) cases. Dashed lines represent a variation of 20%. Coloured dots represent significantly altered metabolites (*P*<0.05). R, recurrent; NR, non-recurrent

Supplemental Table 1. Clinical characteristics of recurrent and non-recurrent EC cases.

		Recurrent									Non-recurrent								
		Age	BMI	Myometrial Invasion	LVSI	Metastasis	Follow-up (Mo)	Radiotherapy	Chemotherapy	Deceased	Age	BMI	Myometrial Invasion	LVSI	Metastasis	Follow-up (Mo)	Radiotherapy	Chemotherapy	Deceased
Type I	Grade 1	71	30.7	> 50%	No	No	60	Yes	No	Yes	63	30.7	> 50%	No	No	86	Yes	No	No
		78	33.5	< 50%	No	No	150	No	No	Yes	63	44.7	< 50%	No	No	59	No	No	Yes
		63	19.9	< 50%	No	No	108	No	No	No	83	22.1	< 50%	Yes	No	23	No	No	Yes
	Grade 2	63	20.6	< 50%	No	No	109	No	No	No	62	18.7	< 50%	No	No	56	No	No	No
		66	23.1	> 50%	No	No	89	Yes	Yes	Yes	60	27.3	> 50%	Yes	No	38	Yes	No	No
		52	22.1	< 50%	No	No	116	No	No	No	54	26.4	< 50%	No	No	62	No	No	No
		67	26.3	> 50%	Yes	Yes	87	No	Yes	Yes	82	26.0	> 50%	No	No	86	Yes	No	Yes
		76	26.8	< 50%	No	No	46	No	No	No	66	27.2	< 50%	Yes	No	55	Yes	No	No
		52	33.4	> 50%	No	No	167	Yes	No	No	67	31.6	> 50%	No	No	62	Yes	No	No
		73	38.1	> 50%	No	No	36	Yes	No	No	69	39.5	> 50%	Yes	No	33	Yes	No	No
		78	32.0	> 50%	Yes	Yes	13	No	Yes	Yes	56	24.7	> 50%	Yes	Yes	94	Yes	Yes	No
	Grade 3	65	40.2	> 50%	No	No	50	Yes	No	Yes	59	39.4	> 50%	Yes	Yes	78	Yes	No	No
Type II	Grade 3	73	19.9	> 50%	No	No	10	Yes	No	Yes	67	22.7	< 50%	No	No	100	No	Yes	No
		69	22.1	< 50%	No	No	21	Yes	Yes	Yes	69	22.7	< 50%	No	No	49	Yes	No	No
		84	23.6	< 50%	Yes	No	23	Yes	Yes	Yes	76	21.0	< 50%	No	No	2	No	Yes	No
		71	28.8	> 50%	Yes	No	29	Yes	No	No	75	26.3	< 50%	No	No	63	Yes	No	No
		50	28.5	> 50%	Yes	Yes	53	No	Yes	Yes	61	28.5	> 50%	Yes	Yes	24	Yes	Yes	No
		64	35.1	> 50%	Yes	Yes	11	Yes	Yes	Yes	61	31.9	< 50%	No	No	43	Yes	Yes	No
